# Dental and temporomandibular joint alterations in rheumatoid arthritis patients and their association with salivary oxidative stress

**DOI:** 10.3906/sag-2102-260

**Published:** 2021-08-30

**Authors:** Deniz YAMAN, Duygu GÖLLER BULUT, Gülbahar USTAOĞLU, Emre AVCI, Murat TAŞÇI

**Affiliations:** 1 Department of Oral and Maxillofacial Surgery, Faculty of Dentistry, Bolu Abant İzzet Baysal University, Bolu Turkey; 2 Department of Dentomaxillofacial Radiology, Faculty of Dentistry, Bolu Abant İzzet Baysal University, Bolu Turkey; 3 Department of Periodontology, Faculty of Dentistry, Bolu Abant İzzet Baysal University, Bolu Turkey; 4 Department of Biochemistry, Faculty of Pharmacy, Gulhane University of Health Sciences, Ankara Turkey; 5 Department of Rheumatology, Faculty of Medicine, Bolu Abant İzzet Baysal University, Bolu Turkey

**Keywords:** Oxidative stress, rheumatoid arthritis, saliva, temporomandibular joint disorders

## Abstract

**Background/aim:**

Rheumatoid arthritis (RA) is the most extensive inflammatory arthritis causing permanent deformities in the joint. Increasing evidence suggests that oxidative stress is a substantial factor in the pathogenesis of RA. This study aimed to examine the salivary oxidant-antioxidant status of RA and control groups and to compare these biomarkers by correlating them with disease activity, acute phase reactants, and clinical findings.

**Materials and methods:**

Age and sex-matched 60 participants including 30 patients with RA and 30 control (50 females, 10 males; mean age: 42.62 ± 10.89 years) were evaluated. RA disease activity and severity were evaluated by the disease activity score 28-C reactive protein (DAS 28-CRP). Rheumatoid factor (RF) positivity, anticitrullinated protein antibodies (ACPA) positivity, erythrocyte sedimentation rate (ESR), CRP, tender and swollen joint counts, and medical treatment regimens of the patients (glucocorticoids, conventional or biologic disease-modifying antirheumatic drugs) were recorded. In the radiographic examination, dental findings, and bone alterations of the temporomandibular joint (TMJ) were recorded and compared for both groups. Saliva samples were obtained for analysis of total antioxidant status (TAS), total oxidant status (TOS), arylesterase (ARE), and oxidative stress index (OSI) levels. The data analysis was conducted by independent sample t-test and chi-square test.

**Results:**

Condylar erosion was the most common radiographic change in TMJ of RA patients. Osteophyte formation was a prominent finding in the control group. Lower TAS and higher OSI levels were found in RA patients compared with controls (p = 0.013; p = 0.029, respectively). The effect of DAS 28-CRP score on the levels of oxidative stress biomarkers in RA patients was not significant.

**Conclusion:**

Oxidative stress causes tissue damage in response to excessive mechanical loading, which in turn promotes TMD. However, disease activity has not a prominent impact on the salivary oxidative stress status of RA patients.

## 1. Introduction

Rheumatoid arthritis (RA) is a progressive, inflammatory and autoimmune disease and makes unfavourable changes in joint structures such as cartilage, tendons, and synovium. These changes lead to chronic pain, tissue edema, motility limitations, decreased health quality/functionality, and accelerated aging. RA can affect many visceral organs or systems too. The major oral cavity findings of RA are progressive tooth loss, xerostomia, and mild to severe temporomandibular joint disorders (TMD) [1,2]. In RA patients with TMD, pain, swelling, crepitation of the joint, and limitation of the mouth opening result from a vicious circle of inflammation [3]. TMD was observed in more than 66% of RA patients, and otalgia, pain in the temporomandibular joint, crepitation, and tinnitus were among the accompanying symptoms [4]. Radiographic findings include erosion and flattening of the condylar head and narrowing of the joint space [5]. 

Although the exact pathology of RA is not known, it has been stated that unbalanced immune activity triggers reactive oxygen species (ROS) production leading to oxidative stress-related microenvironment development in the tissues [6,7]. Indeed, previous studies have shown that lower antioxidant capacity and dense lipid peroxidation metabolites in the serum and synovial fluid were predominant risk factors in the pathogenesis of RA [8,9]. 

Saliva is known as the first antioxidative barrier against oxidative stress [10]. Many enzyme systems such as superoxide dismutase, catalase, glutathione peroxidase constitute the enzymatic protection against ROS [11]. Although there are lots of reports showing functional changes of these antioxidants separately, measuring all of these antioxidants one by one is both time-consuming process and not practical. Therefore, for obvious antioxidant-oxidant homeostasis, total antioxidant status (TAS), total oxidant status (TOS), and oxidative stress index (OSI) which is the ratio of TOS to TAS levels are focuses of current many clinical trials. Unbalanced oxidative stress consumes TAS leading to a low OSI index value [12]. 

Regarding the salivary antioxidants, arylesterase (ARE) requires special attention because the variable activities of ARE are also associated with antioxidative properties [13]. This protein is involved in the oxidation process of lipids and glycose metabolites and is effective in the control of various inflammatory and autoimmune pathways in diseases such as RA [14]. 

To our knowledge, the change in salivary total oxidant/antioxidant status (TOS/TAS/OSI) and ARE activity in RA patients with TMD and controls has not been studied. In line with this, we aimed to investigate the salivary oxidant-antioxidant status of RA and control groups and compare these biomarkers by correlating them with disease activity, acute phase reactants, and clinical findings.

## 2. Materials and methods

### 2.1. Study population 

This cross-sectional research was approved by the local ethics committee (No: 449/2020), and all participants signed a consent form before the start of the study. The study sample consisted of 30 patients recruited randomly in three months (November 2020-January 2021), who are in follow-up at the Bolu Abant İzzet Baysal University, Department of Rheumatology and have symptoms associated with TMD (the feeling of muscular tension or stiffness during the day, masseter, and/or temporal muscle and TMJ pain, restricted mouth opening, and TMJ noises while jaw movement). The age and sex-matched control group were randomly selected among healthy individuals diagnosed with TMD in the Oral and Maxillofacial Surgery Department. Patients with a history of trauma in the maxillofacial region, pregnancy, smoking/alcohol consumption, and antioxidant supplements intake were excluded from the study. 

### 2.2. Clinical assessment

Patients diagnosed with RA according to the 2010 ACR/EULAR classification criteria were included in the study [15]. All of the patients passed on a physical examination. Medical history, drugs, and symptom interrogation were made. Laboratory tests were examined in fasting blood samples. Erythrocyte sedimentation rate (ESR) was assigned using the Westergren technique and C reactive protein (CRP) was determined by nephelometry. Serum rheumatoid factor (RF) was evaluated by the latex agglutination technique. The positivity of antibodies against the cyclic citrullinated peptide (anti-CCP) in serum was determined using the Diastat kit (Axis-Shield Diagnostics, Dundee, UK) with a cut-off value of 5 U/mL. Patients were considered seropositive if any or both of RF or anti-CCP antibodies were positive. At the time of the study, 28 patients were using at least one conventional disease-modifying antirheumatic drugs (csDMARDs) or biologic DMARDs (bDMARDs). Patients were using methotrexate, sulfasalazine, leflunomide, and hydroxychloroquine as csDMARDs. Infliximab and adalimumab were bDMARDs that patients were using. Twenty-one patients (70%) were using glucocorticoids (GC). The mean GC dosage was 3.3 mg/day prednisolone equivalent. Twenty-seven patients were using csDMARDs and six were on bDMARDs. The patients using high doses of GC (>10 mg/day prednisolone equivalent) were excluded from the study to exclude the possible role of GCs enhancing oxidative stress. Disease activity score 28-CRP (DAS 28-CRP) was used for the evaluation of disease activity in RA patients [16]. The total number of swollen and tender joints, CRP values, and patients’ global assessments about disease burden were recorded on a 0–100 mm visual analog scale. DAS 28-CRP ≤ 2.6 was determined as the mean remission, 2.6–3.2 as low disease activity, 3.2–5.1 as moderate, and > 5.1 as high disease activity [16]. 

### 2.3. Assessment of TMJ disorders

Panoramic X-ray and lateral panoramic images were taken for all patients after a detailed and standardized clinical examination. Evaluation of TMD findings in all patients was performed by the same doctor (DY) using the Research Diagnostic Criteria for Temporomandibular Disorders Axis I (RDC/TMD Axis I). RDC/TMD Axis I is a symptom-based system that categorizes common subtypes of TMD according to their physical diagnosis [17,18]. By RDC/TMD Axis I, patients receive the following group diagnosis; muscle disorders (group I); disc displacement (group II); and arthralgia, osteoarthritis, or osteoarthrosis (group III). Group I (muscle disorders) include any pain provoked with palpation or opening associated in the masseter or temporalis muscle. Myofascial pain (Ia) and myofascial pain with limited opening (Ib) are the subgroups of muscle disorders. Disc displacement with reduction (IIa), disc displacement without reduction with limited opening (IIb), and disc displacement without reduction without limited opening (IIc) are among the subgroups of Group II. Group III consists of arthralgia (IIIa), osteoarthritis with osseous joint changes (IIIb), and osteoarthrosis (IIIc). A translation of RDC/TMD into more than 20 languages is an important factor in the widespread use of this index in clinical trials [19]. By using RDC/TMD, we aimed to ensure diagnostic standardization and increase the knowledge about TMD epidemiology by making comparisons between different studies.

### 2.4. Measurement of oxidative stress marker in saliva

To prevent circadian rhythm changes in all patients, a total of 3 mL unstimulated saliva samples were collected between 9:00 and 10:00 in the morning and stored at -80 ° C until analysis of oxidative stress parameters. All patients were instructed to avoid eating or drinking for 1 hour before sampling. The TAS, TOS levels and ARE activity were analyzed spectrophotometrically [5,6,20]. The oxidative stress index (OSI), consisting of the ratio of TOS to TAS, is a crucial marker in this dynamic oxidative network [12], and the analysis of OSI value was made using this formula: 

OSI (arbitrary unit) = TOS (μmol H_2_O_2_ equivalent/L)/TAS (mmol Trolox equivalent/L) [21,22]. 

### 2.5. Statistical analysis

The minimum required sample size has been reckoned using the G*Power software version 3.1.9.4 (Heinrich Heine University, Dusseldorf, Germany). A power analysis was performed based on a previous study [23]. It was estimated that a sample size of 27 participants per group would be 95% effective in detecting a statistically significant difference in oxidative biomarker between the two groups (alpha level = 0.05, effect size = 0.91) [23]. All statistical procedures were evaluated using Statistical Package for Social Science (v. 20.0 SPSS Inc., Chicago, IL, USA). Continuous variables were analyzed using Shapiro–Wilk test for normal distribution. Descriptive statistics were made for some of the data and the results were given as frequency, mean ± standard deviation (SD), minimum and maximum values. An independent sample t-test was used for comparison of the parametric variables. Categorical data were interpreted with the chi-square test. Categorical variables were summarized as counts and percentages. Univariate linear regression models were used to assess the relationship between parameters. The p-value for statistical significance was accepted as < 0.05. 

## 3. Results

Thirty patients in the RA group (25 females, 5 males; mean age: 42.62 ± 10.89 years) and 30 patients in the control group (25 females, 5 males; mean age: 42.62 ± 10.89 years) were included in this study and the collected data was analyzed between RA and control groups. The effect of sex and age on the RDC/TMD Axis I diagnosis was analyzed and it was found to not be statistically significant (for age RA and control group: p = 0.236, p = 0.381; for sex RA and control group, p = 0.598, p = 0.140). 

### 3.1. Analysis of clinical parameters

Clinical characteristics of patients with RA were shown in Table 1. The total follow-up period of patients with RA was 4.97 years. Disease activity in RA patients was assessed by DAS 28-CRP, of which 60% were in remission/low activity and 40% in moderate/high activity. The majority of patients were using csDMARDs (90%) and GCs (70%). Diabetes mellitus (3.3%), hypertension (6.7%), and secondary Sjogren’s syndrome (3.3%) were prevalent co-morbid disorders observed in RA patients in the study.

**Table 1:** Clinical characteristics of patients with rheumatoid arthritis.

Variable	n = 30
Total follow-up (year) [mean (min-max)]	4.97 (0.10–16.0)
CRP [mean (min-max)]	10.78 (0.20–51.00)
ESR [mean (min-max)]	29.53 (8.00–56.00)
DAS 28-CRP [mean (min-max)]	3.05 (1.30–6.20)
DAS 28-CRP < 3.2 (%)	60
DAS28-CRP > 3.2 (%)	40
RF positivity (%)	43.3
ACPA positivity (%)	56.7
Current treatment	
csDMARD (%)	90.0
GC (%)	70.0
bDMARD (%)	20.0
Comorbidities	
Diabetes mellitus (%)	3.3
Hypertension (%)	6.7
Secondary Sjögren’s syndrome (%)	3.3
Others (%)	13.3

C-reactive protein (CRP), the erythrocyte sedimentation rate (ESR), disease activity score 28-C reactive protein (DAS 28-CRP), rheumatoid factor (RF), anticitrullinated protein antibody (ACPA), disease modifying antirheumatic drugs (DMARD), conventional DMARD (csDMARD), biologic DMARD (bDMARD), glucocorticoid (GC), number (n).

### 3.2. Analysis of clinic and radiographic TMJ involvement

According to RDC/TMD Axis I, nine RA patients were diagnosed with myofascial pain (Group Ia), and only 3 RA patients presented disc displacement (Group II). Finally, 18 RA patients were diagnosed with osteoarthritis (Group IIIb). In the control group, 21 patients were in Group Ia, and only 1 patient presented disc displacement and 8 patients were in Group IIIb.

The results from the radiographic changes in the temporomandibular joint (TMJ) showed that RA patients had flattening on the condylar head (13.3%), osteophyte formation (10%), condylar erosion (33.3%), and subchondral sclerosis (6.7%). In the control group, there was flattening on the condylar head (6.7%), osteophyte formation (13.3%), and condylar erosion, and subchondral sclerosis (3.3%). A substantial distinction was detected between groups in terms of radiographic changes in TMJ (p = 0.016 right TMJ; p = 0.022 left TMJ; Table 2). Condylar erosion was more frequent in the RA group compared to the controls. 

**Table 2:** Panoramic radiography findings of rheumatoid arthritis (RA) patients and controls.

	Radiographic changesin TMJ	RA(n = 30)	Control(n = 30)	p* value
Right TMJ	Normal	11 (36.7%)	22 (73.3%)	0.016
	Flattening	4 (13.3%)	2 (6.7%)	
	Osteophyte	3 (10.0%)	4 (13.3%)	
	Condylar erosion	10 (33.3%) *	1 (3.3%) *	
	Subchondral sclerosis	2 (6.7%)	1 (3.3%)	
Left TMJ	Normal	10 (36.7%) *	21 (73.3%)*	0.022
	Flattening	7 (13.3%)	3 (6.7%)	
	Osteophyte	4 (10.0%)	3 (13.3%)	
	Condylar erosion	8 (33.3%) *	1 (3.3%) *	
	Subchondral sclerosis	1 (6.7%)	2 (3.3%)	

number (n), temporomandibular joint (TMJ).* indicates significant difference between RA and control groups.

When the relevance between disease activity and TMJ involvement was evaluated, the effect of DAS 28-CRP scores on the presence of the radiographic change in TMJ was found to be insignificant (p = 0.266). 

### 3.3. Evaluation of denture quality

There was a significant distinction in the number of missing teeth between groups (p = 0.010, Table 3). The number of teeth remaining was higher in the control group and the result was eloquent (p = 0.005, Table 3). The numbers of decayed, filled, root canal-treated teeth and teeth with periapical lesions were compared and no significant results were found between the groups (p > 0.05). Those who use dentures were mostly in RA patients and this relation was found to be meaningful (p = 0.025, Table 3). 

**Table 3:** Dental findings of the participants.

	Rheumatoid arthritis	Control	p* value
The number of teeth remaining (mean ± SD)	22.50 ± 7.065	26.77 ± 3.748	0.005*
The number of missing teeth (mean ± SD)	8.87 ± 7.510	4.70 ± 4.162	0.010*
The number of filled teeth (mean ± SD)	1.87 ± 2.897	2.90 ± 3.111	0.188
The number of root canal treated teeth (mean ± SD)	0.93 ± 1.574	2.00 ± 2.560	0.057
The number of decayed teeth (mean ± SD)	1.37 ± 2.327	1.80 ± 1.955	0.438
The number of teeth with periapical lesions (mean ± SD)	0.47 ± 0.937	0.37 ± 0.850	0.667
No dentures (n)	12	23	0.025*
Removable denture (n)	9	1	
Fixed denture (n)	7	5	
Total and partial dentures (n)	1	0	
Fixed and partial dentures (n)	1	1	

### 3.4. Analysis of oxidative stress biomarkers

While no significant difference was found between ARE activity and TOS levels (p = 0.921 and p = 0.243, respectively), there was a significant distinction in the comparison of TAS and OSI levels between the groups (p = 0.013; p = 0.029, respectively, Table 4). When RA patients were categorized into two groups according to DAS 28-CRP score (below 3.2 and above 3.2) in terms of the activity of the disease, no significant distinction was observed between TAS, TOS levels and ARE activity of both groups (p = 0.508, p = 0.163, and p = 0.955, respectively).

**Table 4:** Comparison of salivary oxidative stress biomarkers between groups.

	Rheumatoid arthritis (mean ± SD)	Control (mean ± SD)	p* value
TAS, mmol/L	0.57 ± 0.25	0.72 ± 0.18	0.013
TOS, µmol/L	14.12 ± 25.37	3.75 ± 1.63	0.243
OSI	2.12 ± 3.15	0.54 ± 0.28	0.029
ARE, U/L	314.20 ± 29.75	318.60 ± 11.44	0.921

Total antioxidant status (TAS), total oxidant status (TOS), arylesterase (ARE), oxidative stress index (OSI), standard deviation (SD).

Table 5 shows the effect of age, drugs, disease duration, and DAS 28-CRP score on oxidant/antioxidant status. According to univariate linear regression analysis, the effect of related parameters on ARE, TOS, TAS dependent variables was insignificant.

**Table 5:** The effect of age, drugs, disease duration, and DAS 28-CRP score on oxidant/antioxidant status.

Parameters		Univariate model	
		B (95.0% confidence interval)	p* value
TAS	bDMARD	–0.127 (–0.369 to 0.115)	0.291
	GC	–0.105 (–0.316 to 0.107)	0.319
	Total follow-up (year)	–0.010 (–0.034 to 0.013)	0,366
	Age	–0.003 –0.012 to 0.007)	0.578
	DAS 28-CRP	–0.006 (–0.207 to 0.196)	0.955
TOS	bDMARD	17.923 (–5.202 to 41.048)	0.124
	GC	3.778 (–24.801 to 17.245)	0.716
	Total follow-up (year)	0.544 (–1.758 to 2.845)	0.632
	Age	0.214 (–0.684 to 1.112)	0.629
	DAS 28-CRP	–6.400 (–25.957 to 13.157)	0.508
ARE	bDMARD	7.667 (–20.493 to 35.826)	0.581
	GC	–9.397 (–33.843 to 15.050)	0.438
	Total follow-up (year)	0.066 (–2.644 to 2.777)	0.960
	Age	0.295 (-0.756 to 1.347)	0.570
	DAS 28-CRP	–15.611 (–37.927 to 6.704)	0.163

Disease modifying antirheumatic drugs (DMARD), conventional DMARD (csDMARD), biologic DMARD (bDMARD), glucocorticoid (GC), disease activity score 28-C reactive protein (DAS 28-CRP), total antioxidant status (TAS), total oxidant status (TOS), arylesterase (ARE), oxidative stress index (OSI).

RA patients were classified according to the duration of the disease. Disease period of ≤3 years were compared those with longer than 3 years [15]. Comparison of oxidative stress and radiographic changes according to the RA disease duration ≤ 3years and >3 years revealed no significant difference.

## 4. Discussion

RA is an inflammatory autoimmune disorder characterized by synovial proliferation, bone destruction, and cartilage degradation. Although the etiology of RA is still unclear, recent studies draw attention to the role of reactive oxygen species in the pathogenesis of the disease. Besides, RA patients have a higher cumulative risk of TMD than the control group, and TMJ symptoms constantly emerged in the early stages of RA [24,25]. The majority of RA-related TMD patients developed symptoms before or shortly within 1 year of the involvement of other body joints [5,24]. In this study, the total follow-up period of patients with RA was 4.97 years and more than half of RA patients belonged to Group IIIb (osteoarthritis). Since the RA history of the patients in our study group is long-term, TMJ findings may be common. Similar to previous studies [26,27], we observed that condylar erosion was the most common pathology in RA with a rate of 33.3% on both sides. The connective tissue degeneration, inflammatory, and degenerative changes of TMJ can be more aggressive and develop faster in RA. However, drugs in RA can control pain, patients may ignore the self-awareness of TMJ problems [28]. The high incidence of condylar erosion can be attributed to this.

Grevers et al. declared that 20% of RA patients suffer from secondary Sjogren’s syndrome [29]. However, our study showed that secondary Sjogren’s syndrome was accompanied by fewer of the patients (3.3%) with RA. This may be because the size of our study population is less than the previous study. Although the salivary glands are remarkably affected in RA, no recent studies are analyzing the antioxidant profile of the salivary secretion of these patients. Hence, the main strength of this study is to research the correlation of biomarkers associated with oxidative stress as well as TMD symptoms and intraoral findings in both groups. 

ARE is an important member of the antioxidant defence system which is reduced in autoimmune diseases [30,31]. Altındağ et al. also reported that the serum ARE activity was lower in RA patients than in the controls [32]. Işık et al. reported that serum ARE activity (359.82 ± 48.94 U/L) in RA patients was significantly lower (393.55 ± 42.27 U/L) than controls [14]. In this study, salivary ARE activity was found as 314.20 ± 29.75 U/L in the RA group and 318.60 ± 11.44 U/L in the control group. Similarly, we observed lower ARE activity in patients with RA than in controls, although this difference was not significant. In this study, the significant high OSI level in the RA group indicates that the TOS level is high and early proactive intervention is required. A significant association between salivary oxidative stress biomarkers with increased oxidative stress products and TMD pain has been previously reported in TMD patients [33]. 

In the present study, the mean TAS value was found to be lower in RA patients (0.57 ± 0.25 (mmol/L) than in the control group (0.72 ± 0.18 mmol/L) (p = 0.013). Nagler et al. stated higher TAS levels for RA patients as 2.17 (mmol/L) than controls as 1.10 (mmol/L) [10]. Almeida et al. reported mean TAS level lower in TMD patients as 0.13 (mmol/L) than in controls as 0.264 (mmol/L) [34]. Similarly, some previous studies gave TAS levels lower in RA or TMD patients [35,36]. Our study also supports the results showing that biomarkers associated with oxidative stress are substantial in the etiology of diseases in the maxillofacial region [14,33]. 

Several clinical studies suggest a possible association between periodontitis/tooth loss and RA [37,38]. Similarly, in these studies, patients with RA had more missing teeth than controls. Also, a significantly higher prosthesis usage point outs the importance of the periodontal status of patients with RA. Over the last decades, the investigators emphasized the importance of oxidative and inflammatory imbalance in patients with poor periodontal health [39,40]. In this study, TMJ damage and osteoarthritic changes have occurred even if the fixed or removable partial denture usage and these treatment applications have no preventive or therapeutic effectiveness on TMD similar to previous studies [41,42].

The limitation of this study was related to the small sample size. Also, the majority of the participants with TMD were females, as stated in the literature [43]. In order to generalize and confirm these results, studies involving more participants are required.

This study is the first to show ARE activity in the saliva of patients with RA and controls. Diminished TAS levels and increased OSI levels were observed in RA patients compared to controls. However, a similarly significant relationship could not be observed at the ARE activity. Besides, disease activity has not a prominent impact on the salivary oxidative stress status of RA patients. Degenerative changes in TMJ can be more aggressive in RA. However, with drugs that control pain in patients with RA, TMJ problems may be overlooked. All these results show the importance of clinical examination and close monitoring for effective detection and early treatment of TMD in RA patients.

## Informed consent

The research was approved by the Ethics Committee of the Bolu Abant İzzet Baysal University with an accession number of 449/2020. All the participants signed a consent form prior to the start of the study.
